# Special Issue “Fracture Mechanics and Fatigue Damage of Materials and Structures”

**DOI:** 10.3390/ma16114171

**Published:** 2023-06-03

**Authors:** Grzegorz Lesiuk, Dariusz Rozumek

**Affiliations:** 1 Department of Mechanics, Materials Science and Biomedical Engineering, Wroclaw University of Science and Technology, Smoluchowskiego 25, PL 50-370 Wroclaw, Poland; 2 Department of Mechanics and Machine Design, Opole University of Technology, Mikolajczyka 5, 45-271 Opole, Poland; d.rozumek@po.edu.pl

## 1. Introduction and Scope

One of the most important aspects of engineering assessment of the technical condition of structures and materials is the ability to assess the fatigue behavior of materials and structures. On the other hand, an important topic is the design of materials or structures to resist fatigue and fracture. Modern science provides us with an increasing number of new materials, from superalloys of metals manufactured conventionally and by additive manufacturing to functionally advanced composites. Against this background, the fundamental knowledge of the fatigue behavior and fracture mechanics of different material groups provides a convenient platform for communication between different interested groups and fields, from material scientists, numerical engineers and mathematical modeling to hybrid methods for fatigue life prediction. This Special Issue provides such an exchange of ideas on recent developments in the field of fatigue and fracture and is especially focused on fatigue crack growth analysis, the description of fatigue damage in metals and composites, probabilistic approaches, fracture mechanics analysis, fatigue failure analysis and lifetime prediction. A summary of the articles is given in this editorial. 

This Special Issue contains thirteen original research papers on fatigue and cracking in materials. The presented articles mainly concern experimental research and numerical calculations. 

## 2. Contributions

In the paper by Volodymyr Hutsaylyuk et al. [1], an analytical–numerical method for determining the mechanical fields in composite structures with interphase ribbon-like deformable multilayered inhomogeneities under combined force and dislocation loading has been proposed. The values of generalized stress intensity factors for the asymptotic stress–strain fields in the vicinity of the ends of thin inhomogeneities are calculated, from which the stress concentration and local strength of the structure can be calculated. The proposed method has shown its effectiveness for solving an entire class of problems of deformation and fracture of bodies with thin deformable inclusions of finite length and can be used for mathematical modeling of the mechanical effects of thin FGM heterogeneities in composites. 

Based on the twin bridge shear specimen, Xinna Liu et al. [2] performed cyclic shear experiments on 1.2 mm thin plates of 316L metastable austenitic stainless steel with different strain amplitudes from 1 to 5% at ambient temperature. The fatigue behavior of 316L stainless steel under the cyclic shear path was studied, and the microscopic evolution of the material was analyzed. The results show that the cyclic stress response of 316L stainless steel exhibits cyclic hardening, saturation and cyclic softening, and the fatigue life is negatively correlated with the strain amplitude. 

The study by Maria Letizia Raffa et al. [3] applies numerical calculations. This paper proposes a numerical assessment of two model parameters, damage energy threshold and damage viscosity, of a hard interface model previously formulated by the authors. The proposed assessment protocol uses macroscopic experimental data, available in the literature, on structural adhesives under standard characterization tests. The numerical results obtained give insights into the physical interpretation of these parameters. 

Furthermore, Krzysztof Junik et al. [4] present the results of a study of polyurethane rigid (PUR) elastomers in terms of constitutive law identification and analyze the effect of polyurethane elastomers’ hardness on fatigue properties. The research objects were PUR materials based on 4,4′-diphenylmethane diisocyanate (MDI) with hardnesses of 80 ShA and 90 ShA, typically used in various industrial applications. Based on the experimental campaign performed under static and cyclic loading, the constitutive model proposed by Ogden is most appropriate. In addition, a hybrid numerical–experimental analysis (using FEM-DIC) of diabolo specimens’ behavior is carried out in fatigue tests. Based on the fatigue test performed, it is worth noting that the energy approach describes the fatigue process synonymously compared to the displacement or strain approach. 

In the article by Antonin Bermond et al. [5], the influence of micro-shrinkage porosity on a G20Mn5 cast steel was presented. G20Mn5 (normalized) ingots were cast under industrial conditions, ensuring the absence of macroporosities. Solidification leads to two very different microstructures prior to the normalization treatment: columnar dendrites beneath the surface (Skin) and equiaxed microstructures close to the center (Core). First, metallographic observations of the whole ingot revealed the same grain size in both areas. Fatigue samples were extracted by differentiating two sampling volumes corresponding to columnar (S) and equiaxed solidification (C), respectively. The distribution of microporosities was determined for all samples by micro-CT scans. Core samples exhibit microporosities with volumes 1.7 times larger than Skin samples. Low-cycle fatigue tests (three levels of fixed plastic strain) were run on both sample series (C, S). The results follow the Manson–Coffin law. Core specimens exhibit a lower fatigue life than Skin specimens. The differences in fatigue life have been successfully related to the differences in microporosity sizes. 

Xi-Ming Yao et al. [6], based on the theory of fracture mechanics, used a finite element method to determine the stress intensity factors of an inclined crack on the inner surface of a pipe under axial compression load and external pressure. The effects of different influencing factors on the stress intensity factor along the crack front were systematically explored considering crack closure and were different from those under internal pressure. The effects of a high aspect ratio on K_II_, the crack inclination asymmetry caused by curvature, and the effects of the friction coefficient on the stress intensity factors of the pipe with an inclined inner surface crack under axial compression load and external pressure were explored. To be suitable for defect assessment, the solutions for stress intensity factors K_II_ and K_III_ were derived, and new correction factors, f_θ_ and f_μ_, were proposed in the empirical solutions to accommodate the crack inclination asymmetry and the friction coefficient, respectively. 

The study by Živilė Decker et al. [7] concerns the durability of suspension components in relation to the duration of the onset of fatigue. This article presents an analysis of damage to the rear axle of the semi-trailer using macroscopic observations of the damage site and dynamic FEA of stress distribution in the axle material. To identify the probable cause of the damage, eight cases of loading the semi-trailer axle were considered. Analytical solutions have shown that in various cases, the yield point is exceeded and the strength limit of the modeled semi-trailer axle is reached. The risk of damage to the vehicle’s suspension system components increases on poor roads (bumps and winding road sections). 

Takayuki Shiraiwar et al. [8] proposed a method for predicting fatigue crack initiation of the 7075 aluminum alloy by crystal plasticity finite element analysis considering microstructures. In order to accurately predict the total fatigue life, it is necessary to calculate the number of cycles for fatigue crack initiation, small crack growth, and long crack growth. The long crack growth life can be estimated by the Paris law, whereas fatigue crack initiation and small crack growth are sensitive to microstructures and difficult to predict. In this work, the microstructure of 7075 aluminum alloy was reconstructed based on experimental observations in the literature, and crystal plasticity simulations were performed to calculate the elasto-plastic deformation behavior in the reconstructed polycrystalline model under cyclic deformation. The calculated local plastic strain was introduced into the crack initiation criterion (Tanaka and Mura, 1981) to predict fatigue crack initiation life. The predicted crack initiation life and crack morphology were in good agreement with the experimental results, indicating that the proposed method is effective in predicting fatigue crack initiation in aluminum alloys. Based on the obtained results, future issues regarding the prediction of fatigue crack initiation were discussed. 

The research by Michał Böhm et al. [9] presented the commonly used EN 1.2709 tool steel (printing steel), which has good strength properties and high abrasion resistance and can be hardened. The research shows, however, that its fatigue strength may differ depending on the printing method and may be characterized by a wide range of fatigue life. Selected S–N curves for EN 1.2709 steel are presented after printing with the selective laser melting method. The characteristics are compared, and conclusions are presented regarding the resistance of this material to fatigue loading, especially in the tension–compression state. A combined general mean reference and design fatigue curve is presented, which incorporates our own experimental results as well as those from the literature for the tension–compression loading state. The design curve may be implemented using the finite element method by engineers and scientists to calculate the fatigue life. 

Faezeh Hatami and Ahmad Varvani-Farahani [10] evaluated the ratcheting response at notch roots of 1045 steel specimens experiencing uniaxial asymmetric fatigue cycles. Local stress and strain components at the notch root were analytically evaluated using Neuber, Glinka, and Hoffman–Seeger (H-S) rules coupled with the Ahmadzadeh–Varvani (A–V) kinematic hardening model. Backstress promotion through a coupled kinematic hardening model with the Hoffman–Seeger, Neuber, and Glinka rules was studied. Relaxation in local stresses on the notched samples as hysteresis loops moved forward with plastic strain accumulation during asymmetric loading cycles was observed. Local ratcheting results were simulated through FE analysis, where the Chaboche model was employed as the material hardening rule. A consistent response of the ratcheting values was evidenced as predicted, and simulated results were compared with the measured ratcheting data. 

In the paper by Branko Nečemer et al. [11], a comprehensive experimental investigation of the high-cycle fatigue (HCF) behavior of the ductile aluminium alloy AA 5083-H111 is presented. The analyzed specimens were fabricated in the rolling direction (RD) and the transverse direction (TD). The HCF tests were performed under load control (load ratio R = 0.1) at different loading levels under a loading frequency of 66 Hz up to the final failure of the specimen. The experimental results have shown that the S–N curves of the analyzed Al alloy consist of two linear curves with different slopes. Furthermore, RD-specimens demonstrated longer fatigue lives if compared to TD specimens. This difference was about 25% at an amplitude stress of 65 MPa, where the average fatigue life was 276,551 cycles for RD specimens and 206,727 cycles for TD specimens. Similar behavior was also found for the lower amplitude stresses and fatigue lives between 106 and 108 cycles. The difference can be caused by large Al6(Mn,Fe) particles that are elongated in the rolling direction and cause higher stress concentrations in the case of TD specimens. The micrography of the fractured surfaces has shown that the fracture characteristics were typical for the ductile materials and were similar for both specimen orientations. 

Similar to [7], Živilė Decker et al. [12] focused on issues related to road transport, which is important for the national economy. Damage usually excludes the means of transport from operation, which causes disruption of supply chains. One such damage is the failure of the suspension system of the vehicle or trailer, which usually occurs when the vehicle is heavily loaded. Such a defective system has been analyzed in this publication. Mathematical apparatus and finite element method (FEM) numerical simulations were used. A dangerous axle cross-section in terms of load was indicated, and the maximum stresses in this area were calculated for two types of roads. On highways, the stress at the critical point was 199 MPa, and on uneven roads, it increased to 304 MPa, which is comparable to the yield point. It was found that the second form of vibration may cause stress in the damage area, but the excitation frequency would have to be quite high. The probability of such a load and failure event occurring under operating conditions is low.

Tong Li et al. [13] studied quasicrystals (QCs), which are representatives of a novel type of material exhibiting many remarkable specific properties. However, QCs are usually brittle, and crack propagation inevitably occurs in such materials. Therefore, it is of great significance to study the crack growth behaviors in QCs. In this work, the crack propagation of two-dimensional (2D) decagonal QCs is investigated by a fracture phase field method. In this method, a phase field variable is introduced to evaluate the damage of QCs near the crack. In the numerical examples, the crack propagation paths of 2D QCs are simulated by the proposed method, and the effects of the phason field on the crack growth behaviors of QCs are studied in detail.

## 3. Conclusions

We would like to thank all of the reviewers for their contributions and efforts in producing this Special Issue, as well as the authors for preparing the papers. We would also like to thank all the staff at the *Materials* Editorial Office, especially Felix Guo, Assistant Editor, who managed and facilitated the publication process.

## References

[1] Hutsaylyuk V., Piskozub Y., Piskozub L., Sulym H. (2022). Deformation and Strength Parameters of a Composite Structure with a Thin Multilayer Ribbon-like Inclusion. Materials.

[2] Liu X., Zhang S., Bao Y., Zhang Z., Yue Z. (2022). Strain-Controlled Fatigue Behavior and Microevolution of 316L Stainless Steel under Cyclic Shear Path. Materials.

[3] Raffa M.L., Rizzoni L., Lebon F. (2022). Numerical Assessment of Damage Parameters for a Hard Interface Model. Materials.

[4] Junik K., Lesiuk G., Duda S., Jamroziak K., Błażejewski W., Zielonka P., Socha T., Denisiewicz A., Kula K., Szczurek A. (2022). Constitutive Law Identification and Fatigue Characterization of Rigid PUR Elastomers 80 ShA and 90 ShA. Materials.

[5] Bermond A., Roume C., Stolarz J., Lenci M., Carton J.-F., Klocker H. (2022). Low Cycle Fatigue of G20Mn5 Cast Steel Relation between Microstructure and Fatigue Life. Materials.

[6] Yao X.-M., Zhang Y.-C., Pei Q., Jin L.-Z., Ma T.-H., He X.-H., Zhou C.-Y. (2023). Empirical Solution of Stress Intensity Factors for the Inclined Inner Surface Crack of Pipe under External Pressure and Axial Compression. Materials.

[7] Decker Ž., Rudzinskas V., Drozd K., Caban J., Tretjakovas J., Nieoczym A., Matijošius J. (2023). Analysis of the Vehicle Chassis Axle Fractures. Materials.

[8] Shiraiwa T., Briffod F., Enoki M. (2023). Prediction of Fatigue Crack Initiation of 7075 Aluminum Alloy by Crystal Plasticity Simulation. Materials.

[9] Böhm M., Niesłony A., Derda S., Owsiński R., Kepka M., Zetkova I., Zetek M., Houdková Š., Prażmowski M. (2023). General Reference and Design S–N Curves Obtained for 1.2709 Tool Steel. Materials.

[10] Hatami F., Varvani-Farahani A. (2023). Accumulation of Plastic Strain at Notch Root of Steel Specimens Undergoing Asymmetric Fatigue Cycles: Analysis and Simulation. Materials.

[11] Nečemer B., Zupanič F., Vuherer T., Glodež S. (2023). High-Cycle Fatigue Behaviour of the Aluminium Alloy 5083-H111. Materials.

[12] Decker Ž., Tretjakovas J., Drozd K., Rudzinskas V., Walczak M., Kilikevičius A., Matijosius J., Boretska I. (2023). Material’s Strength Analysis of the Coupling Node of Axle of the Truck Trailer. Materials.

[13] Li T., Yang Z., Xu C., Xu X., Zhou Z. (2023). A Phase Field Approach to Two-Dimensional Quasicrystals with Mixed Mode Cracks. Materials.

